# Prospective longitudinal study of psychological sequelae, self-perception of body image, and quality of life in severe cutaneous adverse drug reactions: a case-control study

**DOI:** 10.3389/fmed.2026.1774494

**Published:** 2026-05-29

**Authors:** Eddy M. Zitha, Aneliswa Mpungose, Lovemore Mapahla, Avumile Mankahla, Bukiwe Thwala, Mireille Porter, Nadine Teixeira, Jean M. Abrahams, Phuti Choshi, Helen Hoenck, Rhodine Smith, Laura Pirjol, Sipho Dlamini, Elizabeth Phillips, Jonny Peter, Rannakoe J. Lehloenya

**Affiliations:** 1 Division of Dermatology, Department of Medicine, University of Cape Town, Cape Town, South Africa; 2 Division of Allergy and Clinical Immunology, Department of Medicine, University of Cape Town, Cape Town, South Africa; 3 The Modelling and Simulation Hub, Africa, Department of Statistical Sciences, University of Cape Town, Cape Town, South Africa; 4 The Division of Epidemiology & Biostatistics, Faculty of Medicine and Health Sciences, Stellenbosch University, Cape Town, South Africa; 5 Department of Medicine, University of Cape Town, Cape Town, South Africa; 6 Division of Dermatology, Department of Medicine and Pharmacology, Nelson Mandela Academic Hospital, Walter Sisulu University, Mthatha, Eastern Cape, South Africa; 7 Division of Infectious Diseases and HIV Medicine, University of Cape Town, Cape Town, South Africa

**Keywords:** cutaneous body image (CBI) and quality of life (QoL), depression, drug reactions with eosinophilia and systemic symptoms (DRESS), epidermal necrolysis (SJS/TEN), generalized bullous fixed drug eruption (GBFDE), suicide

## Abstract

**Background:**

Severe cutaneous adverse drug reactions (SCARs) are associated with mental health sequelae and reduced quality of life. However, existing research is limited by small retrospective cohorts, overrepresentation of high-income countries, lighter skin tones, inadequate controls, and a paucity of prospective longitudinal case-control studies assessing the psychological sequelae in SCAR.

**Objective:**

This study aimed to prospectively determine and compare the longitudinal trajectories of psychological sequelae in patients with SCAR and controls.

**Methods:**

Between June 2020 and October 2023, SCAR cases and matched controls were recruited. The following validated instruments were used for assessments: (1) the MINI International Neuropsychiatric Interview to assess depression and suicide risk; (2) the Kessler Psychological Distress Scale (K10) to determine pre-existing depression; (3) the Cutaneous Body Image (CBI) scale to measure self-perception of body image; and (4) the Dermatology quality life index (DQLI) to measure the impact of SCAR on health-related quality of life (QoL). Assessments were conducted at admission (baseline or *t*_0_), 6 months (*t*_1_), and 12 months (*t*_2_) after the development of SCARs.

**Results:**

Total of 47 SCAR cases (8 SJS/TEN, 35 DRESS, and 4 GBFDE) and 30 controls were recruited. All had Fitzpatrick skin types ≥III. Cases had significantly higher prevalence of depression [21/47 (45%) vs. 4/30 (13.3′%), *p* = 0.006] and suicide risk [12/21 (57%) vs. 1/4 (25%), *p* = 0.012] compared to controls at *t*_0._ This was maintained at *t*_1_ and *t*_2_. Of 45 cases, 36 (80%) had severe negative self-perception, significantly more than in controls [10/18 (56%), *p* = 0.023] at *t*_0_. Among the cases, 20% continued to experience this negative self-perception, while 9% experienced a deterioration over time. Only one showed persistent negative self-perception among controls. At *t*_0,_ cases had significantly impaired QoL compared to controls_,_ with a median (IQR) DLQI score of [9.3 (4–15) vs. 1.5 (0–3), *p* < 0.001]. However, this effect was lost over time. A major limitation of the study was the high rate of loss-to-follow-up due to coronavirus disease 2019 (COVID-19).

**Conclusion:**

SCARs were found to be associated with a high risk of depression, suicide, impaired QoL, and severe negative self-perception compared to controls. SCAR patients demonstrated persistent or worsening impairments at 6 months and relapses at 12 months. These findings highlight the ongoing psychosocial burden of SCARs and the need for integrated mental health support.

## Introduction

The World Health Organization defines an adverse drug reaction (ADR) as a harmful and unintended response to a drug occurring at normal therapeutic doses (1). ADRs are broadly classified into type A reactions (predictable and dose-dependent), which account for approximately 70–80% of cases, and type B reactions (idiosyncratic and unpredictable), comprising approximately 8–10% of cases (2–4). Severe cutaneous adverse drug reactions (SCARs) are a subset of type B (idiosyncratic) ADRs and represent potentially life-threatening, immune-mediated adverse drug reactions (IM-ADRs) with significant morbidity and mortality, affecting the skin, mucosa, and sometimes internal organs (5).

It is estimated that more than 1 in 10 patients discontinue one or more drugs in combinations used in the treatment of human immunodeficiency virus (HIV), tuberculosis (TB), and other HIV-related co-morbidities due to IM-ADRs (6). IM-ADRs account for one-fifth of ADRs across all populations but occur 10–100 times more commonly in individuals with HIV (7). In South Africa, particularly in HIV- and TB-endemic settings, SCARs are commonly associated with medications used to treat HIV, TB, and opportunistic infections, including antiretrovirals (such as nevirapine, efavirenz, and abacavir), first-line anti-TB drugs, and antibiotics such as sulphonamides, dapsone, and fluconazole (7–9). In contrast, in Asian and Caucasian populations, allopurinol is a leading cause of Stevens–Johnson syndrome/toxic epidermal necrolysis (SJS/TEN) and drug reaction with eosinophilia and systemic symptoms (DRESS), alongside antibiotics (*β*-lactams, sulfonamides, vancomycin, and fluoroquinolones), anticonvulsants (such as carbamazepine and lamotrigine), and non-steroidal anti-inflammatory drugs (NSAIDs). These differences reflect variation in drug exposure patterns and underlying genetic susceptibility across populations (10–13). The incidence of SCARs is reported to be as high as 13% with first-line anti-TB drugs and 8% with NNRTIs (14).

The spectrum of SCARs includes epidermal necrolysis (SJS/TEN), generalized bullous fixed drug eruption (GBFDE), DRESS, and acute generalized exanthematous pustulosis (AGEP) (5, 15).

SJS and TEN form a clinical spectrum referred to as SJS/TEN. They are characterized by pan-epidermal necrosis of the skin and mucosa, along with a positive Nikolsky sign. In SJS, there is ≤10% epidermal detachment, while in TEN, the detachment is ≥30%. SJS-TEN overlap lies between these two extremes (16). SJS/TEN is linked to significant morbidity and mortality. The in-hospital mortality rate can reach as high as 29.5%, with varying rates across the spectrum: approximately 5–10% in SJS, 10–25% in SJS/TEN overlap, and 25–50% in TEN, with significant medium- to long-term sequelae (17–19). A similar but typically less severe reaction is generalized bullous fixed drug eruption (GBFDE). This condition is characterized by more than 10% body surface area (BSA) involvement and extensive vesicular lesions that are confluent and bullous. Although these lesions may resemble those seen in SJS/TEN, GBFDE typically lacks the characteristic involvement of ≥2 mucosal surfaces, which is a defining feature of SJS/TEN (16, 20–22). The GBFDE-associated mortality rate, although generally reported to be lower than that of SJS/TEN, is similarly high at 22%. However, the GBFDE mortality rate is particularly high in older patients (20). Drug reactions with eosinophilia and systemic symptoms (DRESS) are, by definition, associated with internal organ involvement. Its clinical features include a typical rash, fever (>38 °C), eosinophilia, atypical lymphocytosis, lymphadenopathy, and dysfunction of internal organs. Depending on variables such as age and comorbidities, reported mortality rates can be as high as 10%. DRESS are also associated with a spectrum of short- to long-term sequelae including internal organ failure, autoimmunity, and mental health disorders (23–25). On the other hand, acute generalized exanthematous pustulosis (AGEP) is typically regarded as having a less severe clinical course than SJS–TEN and DRESS, with a mortality rate of less than 5% and an incidence of 1–5 cases per million per year (26, 27). It presents with sterile pustular eruptions localized within the epidermis (28). Patients with AGEP may also exhibit systemic features, including fever (>38 °C), leukocytosis (>10,000 cells/mm^3^), neutrophilia (>7,000 cells/mm^3^), eosinophilia, hepatic dysfunction, renal impairment, acute respiratory distress syndrome, and lymphadenopathy (29, 30). The onset of AGEP is typically rapid, occurring within 24–48 h after the initiation of the causative drug (31).

SCARs have a profound impact on quality of life (QoL) of patients, leading to emotional distress, psychological stress, and anxiety. An American survey found 53% of adult inpatients with SJS/TEN experienced depression, 43% reported anxiety, and 20% had post-traumatic stress disorder. Two-thirds were afraid of taking new medications (32). In a recent study involving 24 SJS/TEN cases, we found that 65% had depressive symptoms, with those having TEN experiencing more symptoms (25). A British study found 78% of SJS/TEN patients to be depressed and anxious, with 46% experiencing nightmares, intrusive thoughts, and flashbacks (33). Similarly for DRESS, our study found that 20% of the participants had persistent depressive symptoms and anxiety symptoms 6-month after hospital discharge (25). However, there is a lack of data on the psychological impact of GBFDE. Additionally, there is limited information regarding the psychological impact of SCARs in high HIV/TB settings and among younger cohorts with darker skin tones. Recent studies on SCARs have limitations, such as a small sample sizes, lack of ideal control groups, and observer bias (32, 34–37). These limitations impact the validity and applicability of their findings.

To address these gaps, a case-control study was conducted to assess the psychological sequelae and burden of SCARs among HIV and TB-infected South African patients, with clearly defined and appropriately matched controls, enabling more robust evaluation of potential associations while minimizing bias. Furthermore, this study hypothesized that patients with the most severe forms of SCARs, such as SJS/TEN, DRESS, or GBFDE, are more likely to develop depression, are at a higher risk of suicide, exhibit poor self-body image, and have poor QoL compared to those without these conditions over a 12-month period. This study aimed to prospectively determine and compare the longitudinal trajectories of psychological sequelae in patients who developed SCARs (cases) compared to controls.

## Materials and methods

### Study design

The study was a prospective longitudinal case-control study that was conducted between 1 June 2020 and 30 October 2024 at the tertiary dermatology/drug allergy clinics of the University of Cape Town (UCT) and Walter Sisulu University (WSU). Both institutions have extensive experience in providing non-ICU based supportive care for SCARs, which reflects the level of care delivery in many other lower-middle-income country (LMIC) settings across Africa.

The study was approved by the Institutional Ethics Committee (HREC Ref: 270/2020) as a subset of the existing prospective Immune-mediated Adverse Drug Reactions in African TB HIV endemic settings (IMARI) Registry and Biorepository: WSU (HREC: 056/2020) and UCT (HREC R031/2018) (38). The study involved comprehensive prospective follow-up of all eligible SCAR patients admitted to the study sites, with assessments conducted at 6-month intervals from the initial 6-week follow-up for up to 1 year post-IM-ADR. The psychological outcomes of SCAR patients was compared with those with controls matched for age, gender, HIV status, and cluster of differentiation 4 (CD4) T-cell count. To assess the potential attrition bias, baseline demographic and clinical characteristics were compared between participants retained at 6 months and at 12 months and those lost to follow-up using the logistic regression models. We performed subgroup and sensitivity analyses to separate controls with dermatological conditions (those with skin diseases) from controls with non-dermatological conditions (those without skin diseases).

### Inclusion and enrolment

All cases of SCARs prospectively enrolled to the IMARI registry were asked whether they were interested in participating in this longitudinal sequelae (physical and neuropsychological) sub-study. Participants who expressed interest provided additional consent for this sub-study. The IMARI Registry and Biorepository includes participants aged over 12 years with a confirmed SCAR diagnosis as validated by a dermatologist or aged- and gender-matched drug-tolerant controls. Written informed consent was obtained from parents or legal guardians, with age-appropriate assent from minors, where applicable. The consent process was conducted by trained research officers in accordance with ethical guidelines. Cases were defined as participants with a dermatologist-confirmed diagnosis of SCARs, while controls were age- and gender-matched participants with documented tolerance to the suspected drugs and no history of SCARs. They were further subdivided into individuals with skin disease and those without skin disease.

SCAR cases included phenotypes such as SJS/TEN, DRESS, and GBFDE, as defined using the RegiSCAR or consensus case criteria (39–41). Additionally, the registry included participants with other phenotypes classified as controls. These comprised non-SCARs including morbilliform eruption (MBE), lichenoid drug reaction (LR), fixed drug eruption (FDE), transient reactions (TR), drug-induced lupus (DIL), and a drug-tolerant group (DTG), which consisted of participants who had completed at least 8 weeks of therapy without developing IM-ADRs. These controls were recruited in a similar prospective manner to cases. All participants were enrolled at baseline (from 0–6 weeks post-onset of acute SCAR), and follow-up contacts were attempted at 6 months and 12 months.

### Data collection

Baseline data consisted of demographic information (including age, gender, and self-reported ethnicity) and relevant clinical data relating to SCAR phenotype and controls, extracted from the IMARI registry. These include: HIV/CD4 count and TB status (present status and treatment details), duration of hospitalization, Fitzpatrick skin tone, and body surface area (BSA) involvement in SCARs. BSA assessments were conducted by qualified dermatologists who reviewed clinical photographs of each participant and the acute event, using the Rule of Nines to estimate the total percentage of epidermal detachment. This included blistering, positive Nikolsky areas, epidermal necrosis, and erythematous but intact skin, based on standard regional.

Psychological sequelae were defined as newly developed or persistent depression, suicidal risk or behavior, reduced QoL related to emotional well-being, and negative body image perception due to SCAR, observed during hospitalization and extending beyond 6 weeks using validated tools. The following instruments were used, included:

1. The Mini-International Neuropsychiatric Interview (M.I.N.I.) is a validated, structured, clinical interview designed to diagnose psychiatric disorders according to the Diagnostic and Statistical Manual of Mental Disorders, Fourth Edition (DSM-IV) or International Classification of Diseases, 10th Revision (ICD-10) in epidemiological studies, multicentre clinical trials, and non-research clinical settings. This instrument comprises of modules for 17 psychiatric diagnoses and requires “yes” or “no” responses (42).
2. The Kessler Psychological Distress Scale (K10) is a validated scale that measures psychological distress. It determines the likelihood of having a mental disorder (psychological distress) prior, and comprises of 10 questions about emotional states, each with a five-level response scale. Items are scored from 1 (“none of the time”) to 5 (“all of the time”), yielding a minimum score of 10 and a maximum score of 50. Low scores indicate low psychological distress, while high scores indicate severe psychological distress (43, 44).
3. The cutaneous body image (CBI) is a psychometrically validated scale used in both Canadian and Japanese dermatology samples and non-clinical participants (45, 46). The scale is designed to assess patient’s self-evaluative perceptions of the appearance of skin, hair, and nails. It consists of 7 items, in which subjects are required to answer, “not at all”, “slightly”, “moderately”, “Markedly”, and “very markedly” denoted by a scale of 0–9, indicating the strength of agreement with each item and average of all 7 ratings is reported as score. A score <3 is regarded as severe, a score between 3 and 6 is regarded as moderate, and a score >6 indicates a mild to none (45, 46).
4. This study conducted the MINI to screen and diagnose depression and suicide risk levels, while the K10 assessed the pre-existing depression during the 4 weeks prior to admission. In addition, both the cutaneous body Image scale (CBIS) and the Dermatology quality life index (DQLI) were administered to measure the impact of SCAR on participant’s health-related QoL and self-perception of body image. These were conducted at each time point, such as baseline (*t*_0_), 6-month (*t*_1_), and 12-month (*t*_2_). Participants reporting suicide risk at any point during the study were referred to a psychiatrist. All questionnaires were administered in the participants’ preferred language.

### Statistical analysis

Descriptive statistics with percentages, median, and interquartile range (IQR) were used, and used the Shapiro–Wilk testing to determine normality. The Chi-square and the Mann–Whitney tests were used for univariate comparisons between cases and controls for categorical and continuous variables, respectively. Independent *T*-test were used to test duration of admission mean difference between cases and controls. The Fisher’s exact test was applied to compare depression and suicide risk levels, CBI scores, and DQLI scores between cases and controls (*p* < 0.05) at baseline, 6-month, and 12-month follow-up visits. A multivariable logistic regression model was used to assess predictors of depression, CBI (<3), and DLQI scores (≥6), adjusted for age, gender, HIV status, TB status, and admission days. All statistical tests were conducted at a 5% level of significance. Stata programming language (version 15) was used for all data analyses.

## Results

### Demographic and characteristics of the study population

A total of 213 participants were initially enrolled (124 cases and 89 controls). Of these, 82 (47 cases and 35 controls) did not meet the inclusion criteria and were excluded, and 54 (30 cases and 24 controls) were lost to follow-up. The final sample included 77 participants (47 cases and 30 controls), representing a 36.1% overall retention rate (Table 1). Attrition rates were comparable between cases (37.9%) and controls (33.7%) (see Supplementary Table S1). Loss-to-follow-up at 6 months differed significantly between cases and controls (*p* = 0.011) and was associated with attrition (*p* = 0.024), indicating differential attrition. However, baseline age, gender distribution, and baseline depression did not differ significantly between participants retained in the study and those lost to follow-up (all *p* > 0.05). Subgroup analysis assessing control group heterogeneity showed that those with skin disease among controls were significantly associated with case status (*p* = 0.012), whereas no significant association was observed for those without skin disease (see Supplementary Tables S2, S3). Among the cases, 35 (75%) were DRESS, 8 (17%) SJS/TEN, and 4 (8%) GBFDE.

**Table 1:** Demographic and characteristics of 47 SCAR cases and 30 controls analyzed.

Variable	Overall, *n* = 77	Cases, *n* = 47	Control, *n* = 30	*P-*value
Cases, *n* (%)				
DRESS	35 (75%)	35 (75%)	0 (0.0)	
SJS/TEN	8 (17%)	8 (17%)	0 (0.0)	
GBFDE	4 (8%)	4 (8%)	0 (0.0)	
Controls, *n* (%)				
*Without skin disease, n = 8*				
DILI	1 (4.3%)	0 (0.0)	1 (4.3%)	
TB drug-tolerant controls	7 (23%)	0 (0.0)	7 (23%)	
*Skin disease, n = 22*				
LDR	6 (26%)	0 (0.0)	6 (26%)	
MBE	3 (13%)	0 (0.0)	3 (13%)	
TR	3 (13%)	0 (0.0)	3 (13%)	
FDE	3 (13%)	0 (0.0)	3 (13%)	
Eczema	1 (4.3%)	0 (0.0)	1 (4.3%)	
DIL	1 (4.3%)	0 (0.0)	1 (4.3%)	
ICD	1 (4.3%)	0 (0.0)	1 (4.3%)	
SCAR-UP	4 (17.4%)	0 (0.0)	4 (17.4%)	
Offending drugs, *n* (%)				
Antibiotic [trimethoprim–sulfamethoxazole (Bactrim)]	29/77 (38%)	22/47 (46.8%)	7/30 (23.3%)	0.038*
Anti-tuberculosis (Rifafour)	33/77 (43%)	18/47 (38.3%)	15/30 (50.0%)	0.312
Anticonvulsants	4/77 (5.2%)	4/47 (8.5%)	0/30 (0.00%)	0.101
Antiretrovirals	6/77 (7.8%)	0 (0.00%)	6/30 (20.0%)	0.0014*
Other drugs (allopurinol and NSAIDs)	10/77 (13%)	8/47 (17.0%)	2/30 (6.7%)	0.1898
Age in years, median (IQR)	40 (33–51)	40 (34–51)	36 (30–53)	0.558
Gender, *n* (%)				
Women	47 (61)	28 (60)	19 (63)	0.742
Men	30 (39)	19 (40)	11 (37)	
Self-reported ethnicity, *n* (%)				
Black	55 (71.43)	34 (72.34)	21 (70)	0.816
South African colored	17 (22.08)	9 (19.15)	8 (27)	
Indian	1 (1.30)	1 (2.13)	0 (0.0)	
White	4 (5.19)	3 (6.38)	1 (3)	
HIV, *n* (%)				
Negative	20 (25.97)	12 (25.53)	8 (26.67)	0.912
Positive	57 (74 0.03)	35 (74.47)	22 (73.33)	
CD4 count, median (IQR), *n* = 56	117 (54–230.5)	115 (66–181)	158 (45–315)	0.370
TB, *n* (%)				
No	29 (37.66)	13 (27.66)	16 (53.33)	0.023*
Yes	48 (62.34)	34 (72.34)	14 (46.67)^**^	
TB/HIV co-infection, *n* (%), *n* = 63				
No	21 (33.33)	5 (13.51)	16 (61.54)	<0.001*
Yes	42 (66.67)	32 (86.49)	10 (38.46)^†^	
BSA of rash, median (IQR)	60 (18–80)	70 (40–85)	22.5 (0–70)^‡^	<0.001*
Admission days, median (IQR)	26.30 (46.14)	28.43 (21.25)	22.97 (69.6)	0.616
Mortality rate, *n* (%)	9 (11.7%)	8 (17%)	1 (3.3′%)	0.068

*n* (%), number of participants and percentages; IQR, interquartile range; LDR, Lichenoid drug reaction; MBE, morbilliform eruption; FDE, fixed drug eruption; TR, transient reactions; DIL, drug-induced lupus; ICD, irritant contact dermatitis; DILI, drug induced liver injury; SCAR-UP, uncharacterized phenotype; HIV, human immunodeficiency virus; TB, tuberculosis; ^**^ TB = This applies to the TB-diseased group and to the skin disease group with concurrent TB; ^†^TB/HIV co-infection = This applies to skin disease group; ^‡^BSA = This applies to skin disease group only; BSA, body surface area; Because age and CD4 count are normally distributed, this study utilized the median (interquartile ranges) to describe it. The rest of the results were presented as totals with percentages; * *P*-value <0.05 = statistically significant between cases versus controls.

The controls group comprised participants without skin disease (8/30, 27%) including 1 drug-induced liver injury (DILI), 7 (23%) TB drug-tolerant participants (with normal skin), and 23 (77%) participants with skin disease. Among those with skin disease, 6 (20%) LDR, 3 (10%) MBE, 3 (10%) FDE, 3 (10%) TR, 1 (3.3%) eczema, 1 (3.3%) DIL, 1 (3.3%) irritant contact dermatitis, and 4 (13.3%) uncharacterized SCAR phenotype. Across phenotypes, antibiotics and anti-tuberculosis drugs were the most frequently implicated agents. In DRESS (*n* = 35), trimethoprim–sulfamethoxazole (Bactrim, 42.9%) and anti-tuberculosis drugs (40.0%) predominated, with smaller contributions from anticonvulsants (11.4%) and other agents (20%). In EN (*n* = 8), antibiotics were most common (62.5%), followed by anti-tuberculosis drugs (37.5%), while GBFDE (*n* = 4) showed equal distribution between these groups (50% each). Among other phenotypes (*n* = 30), LDR were most frequent, with anti-tuberculosis drugs (46.7%) and antibiotics (20%) as the causative agents. Drug-tolerant controls were primarily exposed to antiretroviral therapy and Rifafour (RHZE) without adverse reactions.

Antibiotics, particularly Bactrim, were more frequently implicated in cases than controls [22/47 (46.8%) vs. 7/30 (23.3%), *p* = 0.038], whereas anti-tuberculosis drugs were more common in controls [18/47 (38.3%) vs. 15/30 (50.0%), *p* = 0.312]. Anticonvulsants and other high-risk agents were observed predominantly in cases [4/47 (8.5%) vs. 0/30 (0.00%), *p* = 0.10], while antiretrovirals were mainly reported in controls [0 (0.00%) vs. 6/30 (20.0%), *p* = 0.068].

The median (IQR) age was 40 (34–51) years for cases and 36 (30–53) years for controls. Women comprised 28 (60%) of cases and 19/30 (63%) of controls. The majority of participants were HIV-positive (75 and 73% of cases and controls, respectively), with 32/47 (69%) of cases and 10 (39%) of controls co-infected with HIV and TB. There was no significant difference in age (*p* = 0.5583), gender (*p* = 0.742), self-reported ethnicity (*p* = 0.816), HIV status (*p* = 0.912), CD4 count (*p* = 0.3697), or duration of hospitalization in days (*p* = 0.616) between cases and controls. However, there was a significant difference in TB status (*p* = 0.023), HIV/TB co-infection (*p* < 0.001), and BSA (*p* < 0.001). A total of 9 (11.7%) deaths were recorded, including 6 among DRESS cases, 2 among SJS/TEN cases, and 1 LDR from the control group. Table 1 details the characteristics of the 77 participants who were enrolled in the study. Each detailed characteristic of the 77 participants is summarized in Supplementary Table S4.

**Figure 1:**
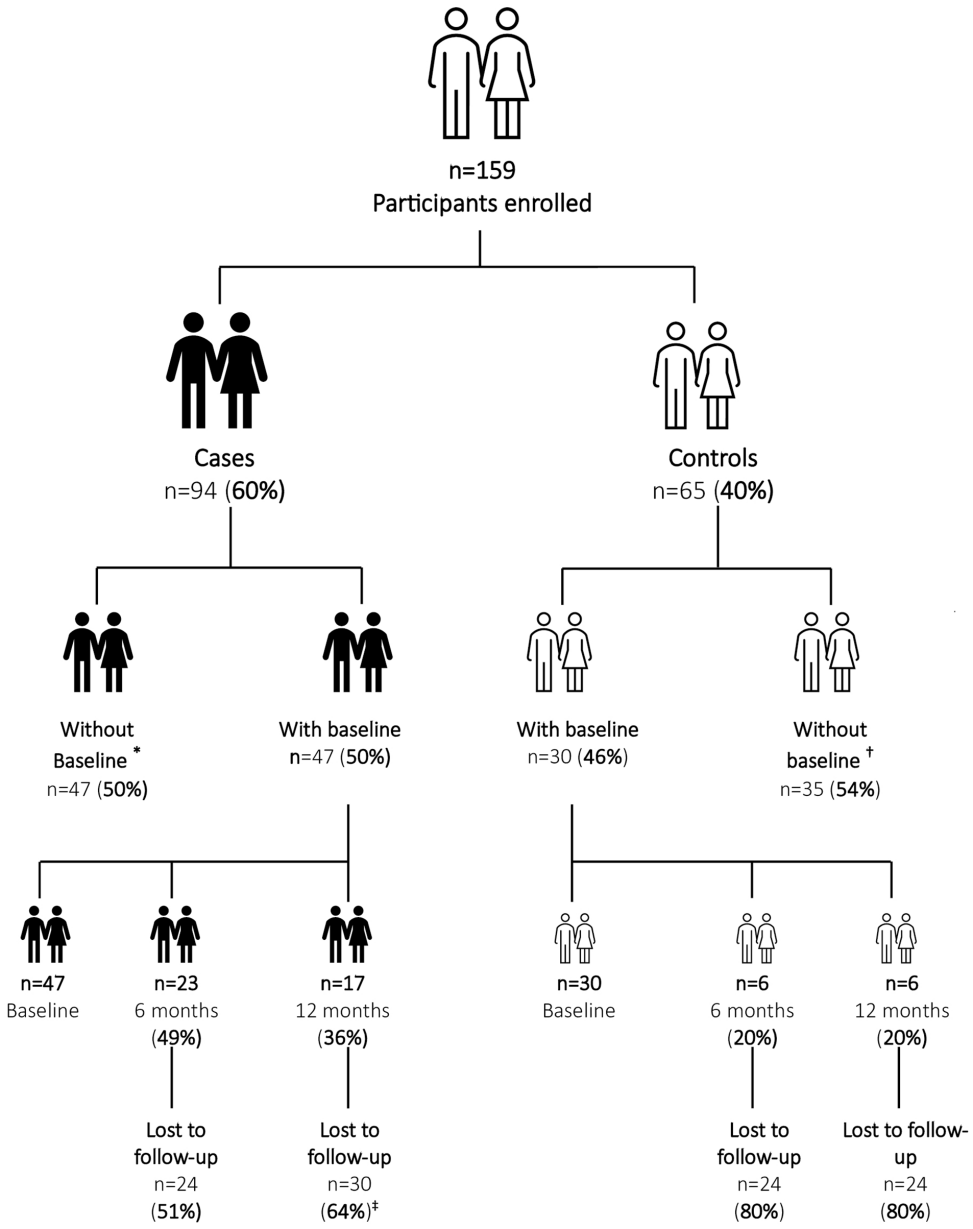
Participants enrolled in the study. * Case group without baseline = cases identified retrospectively were excluded from the analysis. † Control group without baseline = cases identified retrospectively were excluded from the analysis. ‡ This includes all previously lost participants and additional 5 who were unreachable.

A total of 24 cases were lost to follow-up at 6 months and 30 at 12 months. Among controls, 24 participants were lost to follow-up at both at 6 months and 12 months (see Figure 1).

### Comparative analysis of depression and suicide risk between cases and controls at baseline, 6-month, and 12-month follow-up

Pre-existing depression in the 4 weeks prior to admission, assessed with K10: 19/21 cases (90%) and 3/4 controls (75%) were depression-free; 2 cases had mild to severe symptoms, and 1 control had mild symptoms.

At baseline, 25/77 (33%) participants met criteria for depression, of whom 13/25 (52%) had suicide risk. Cases had significantly higher prevalence of depression [21/47 (45%) vs. 4/30 (13.3%), *p* = 0.006] and suicide risk [12/21 (57%) vs. 1/4 (25%), *p* = 0.012] compared with controls.

At 6-month follow-up, depression persisted in only 4/23 (17%) cases and in no controls (*p* = 0.024), with significant 2/4 having ongoing suicide risk, similarly higher than controls (*p* = 0.023). Among cases with baseline depression, 9/23 (39%) achieved remission at 6 months, while 1/23 (4%) developed a new onset of depression. There were 24/21 (51%) cases or controls lost to follow-up.

**Figure 2:**
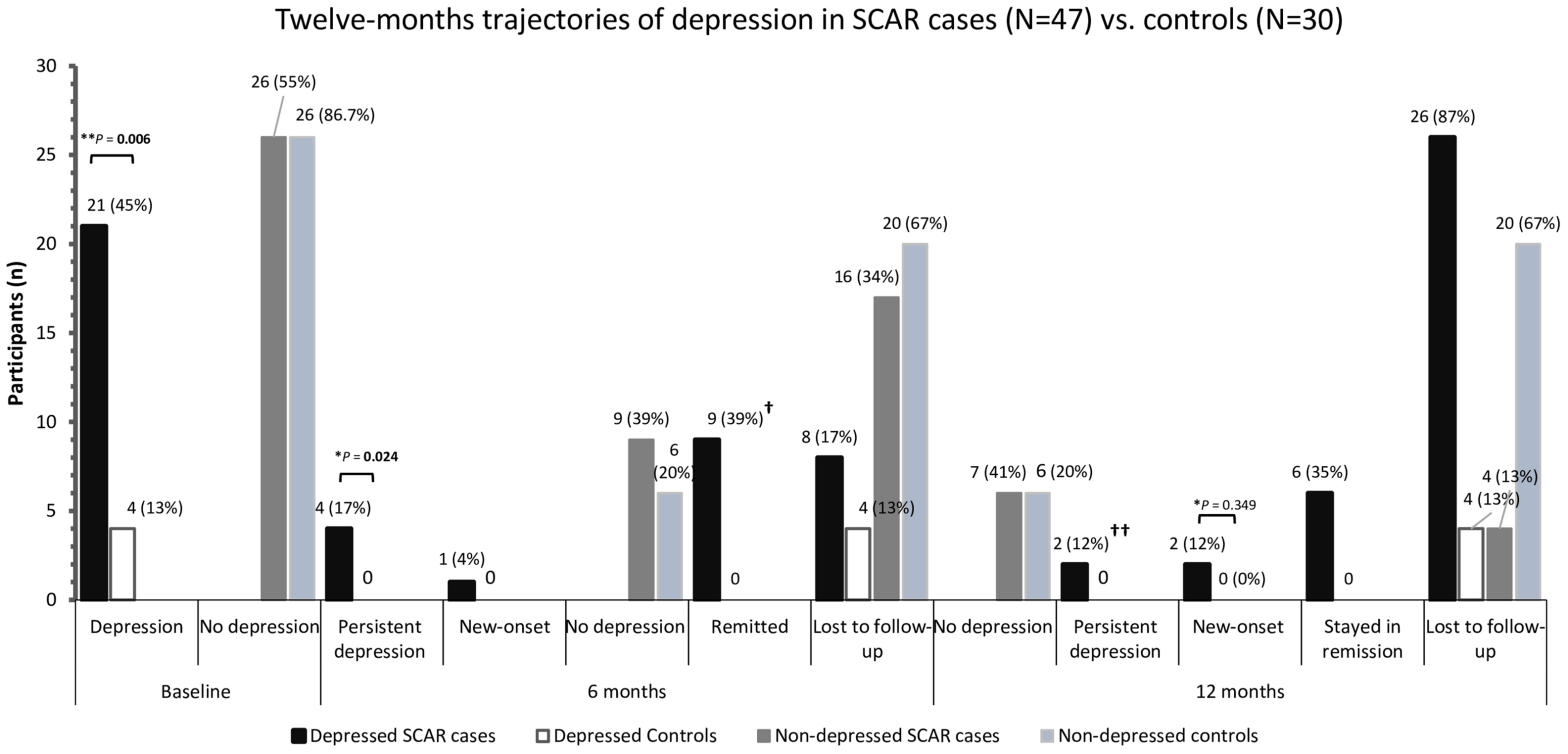
Depression trajectories in SCAR cases vs. controls across 12 months; *N*, total number of participants and (%) percentages; *n*, number of participants; SCAR, severe cutaneous drug reactions; persistent depression → still meets criteria; remitted/in remission → previously met diagnostic criteria for depression but no longer meets those criteria at follow-up; new-onset → no depression at baseline, but developed depression for the first time during follow-up; relapsed →depression at baseline, then went into remission at 6/12, but later redeveloped depression during 12/12 follow-up; † (SCAR-participants that remained without depression both at 6 months and 12 months); †† (one participants with depression at baseline was lost to follow-up at 6 months and remained depressed at 12 months were classified as having persistent depression, given that remission status during the missing interval could not be established) * *p*-value <0.05, ** *p*-value <0.01 statistically significant in depression status in cases vs. controls outcomes at baseline, 6 months, and 12 months follow-up.

At 12-month follow-up, 4/17 (24%) cases met criteria for depression, with (3/4) 75% having suicide risk. There were no significant difference cases and controls [2/17 (12%) vs. 0/4 (0%), *p* = 0.340], with no significant difference in suicide risk (*p* = 0.347). Among the four cases meeting depression criteria: 2/4 (50%) of cases without depression from baseline developed new onset of depression. One case participant (25%) with depression at baseline was lost to follow-up at 6 months and remained depressed at 12 months was classified as having persistent depression from baseline, given that remission status during the missing interval could not be established. Six cases (35%) stayed in remission, while one relapsed. A total of 26 (87%) cases were lost to follow-up, including 4/7 cases (13%) without depression, and all controls (Figure 2). The odds of depression among cases compared with controls were 86% higher at baseline (aOR: 1 vs. 0.14; 95% CI: 0.03–0.55; *p* = 0.005). Depression was significantly associated with high DQLI scores at both baseline (*p* = 0.005) and 6-month (*p* = 0.03). Each additional admission day showed a trend towards lower odds of depression, but the association was not statistically significant (aOR, 0.99; 95% CI, 0.98–1.01; *p* = 0.53).

### Comparative analysis of self-perception of body image (CBI scores) between cases versus controls at baseline, 6-month, and 12-month follow-up

**Figure 3:**
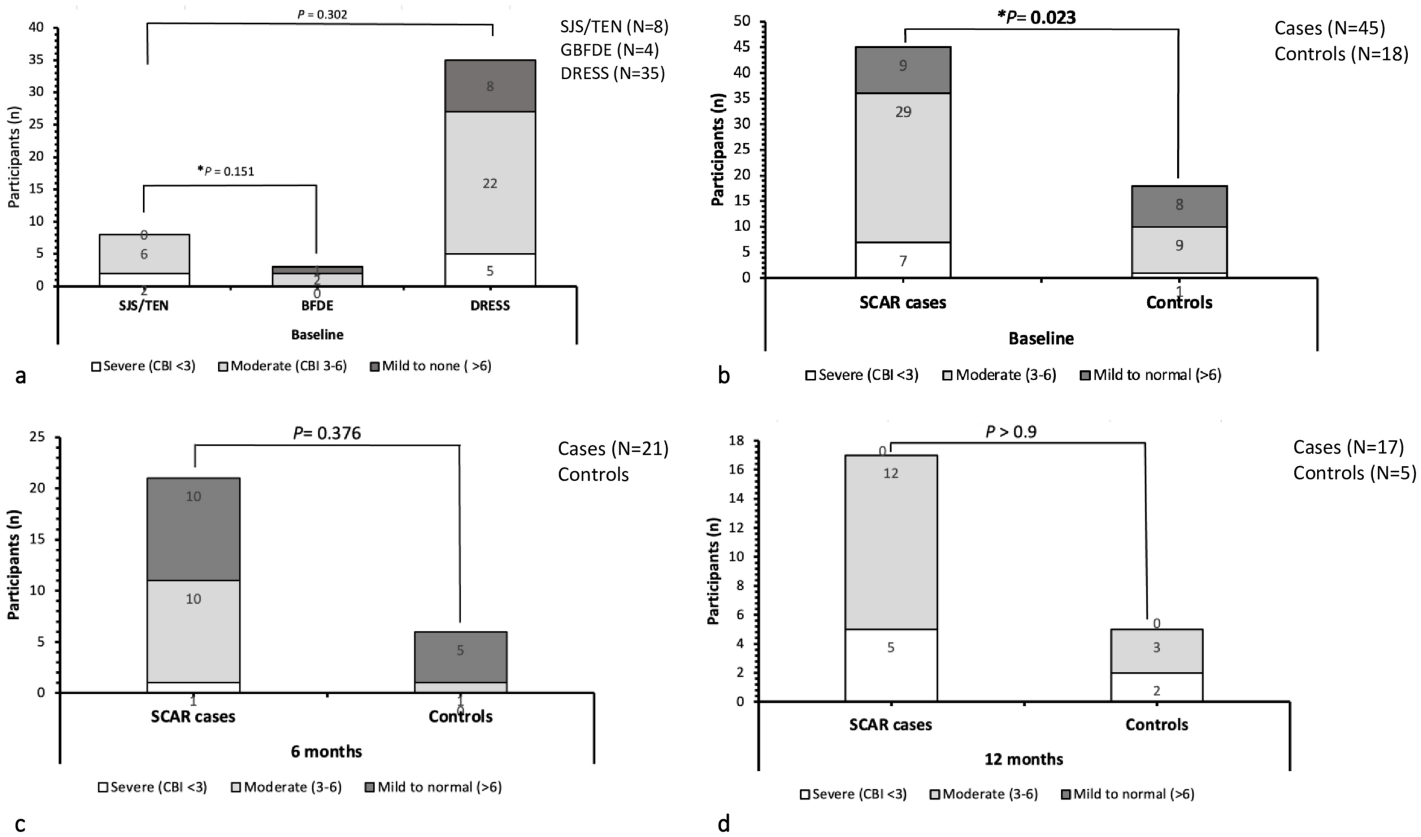
Disease severity distribution of self-perception of body image in cases and controls among phenotypes at baseline **(a)**; cases vs. controls at baseline **(b)**; cases vs. controls at 6 months **(c)**; cases vs. controls at 12 months follow-up **(d)**.

At baseline, 63/77 (82%) participants completed the CBI questionnaire. There was no significant difference in the distribution of CBI scores across the three phenotypic SCAR groups. A one-way ANOVA showed no significant differences between phenotypes (*p* = 0.497). The post-hoc Bonferroni comparisons similarly demonstrated no significant pairwise differences: SJS/TEN versus DRESS (*p* = 0.302) and SJS/TEN versus GBFDE (*p* = 0.151) (Figure 3a). Among cases, 9/45 (20%) were classified as mild to normal, 29/45 (64%) as moderate, and 7/45 (16%) as severe. In contrast, the majority of controls fell within the mild category 8/18 (44%), 9/18 (50%) moderate, and only 1/18 (6%) severe (see Figure 3b). When comparing the CBI score (<6) outcomes between the cases and controls, there was a statistically significant difference (*p* = 0.023). Among the cases, 36/45 (80%) fell into the affected group (moderate/severe) compared to 10/18 (56%) of the controls. The odds of severe CBI (<3) in cases compared with controls was 48% higher at baseline (aOR, 1.00 vs. 0.52; 95% CI, 0.12–2.81; *p* = 0.386), although this difference was not statistically significant. The odds of severe CBI (<3) among participants with baseline depression were 70% higher (aOR, 1.00 vs. 1.70; 95% CI, 0.46–6.27; *p* = 0.423) compared with participants without baseline depression also not statistically significant difference.

At 6-month follow-up, among cases, 10/21 (48%) were classified as mild to normal, 10/21 (48%) as moderate, and 1/21 (5%) as severe. In contrast, the majority of controls fell within the mild category 5/6 (83%), with 1/6 (17%) moderate and none severe (see Figure 3c). Overall, 52% of the cases fell into the affected group (moderate/severe) compared with 1/6 (17%) of controls. This difference was not statistically significant (*p* = 0.376). From baseline, among the cases, 8/36 (22%) achieved remission by 6 months, 4/36 (11%) had persistent CBI impairment, 4/36 (11%) experienced worsening CBI, and 1/36 (3%) had partial improvement. Fifty-three percent of the cases were lost to follow-up. In contrast, among the controls, the majority lost to follow-up and only 3/10 (30%) achieved remission (see Figure 3e).

At 12-month follow-up, 4/17 (24%) % cases fell into the affected group (moderate/severe) compared with 1/5 (20)% of controls. Most controls had scores within the unaffected range (normal/mild). This difference was not statistically significant (*p* > 0.9; Figure 3d). Among the cases, 2/4 (50%) had persistent CBI impairment, 1/4 (25%) relapsed, and only 1/5 (20%) relapsed from controls (see Figure 3e).

Comparative analysis of dermatology quality of life (DLQI scores) between cases and controls at baseline, 6-month, and 12-month follow-up.

**Figure 4:**
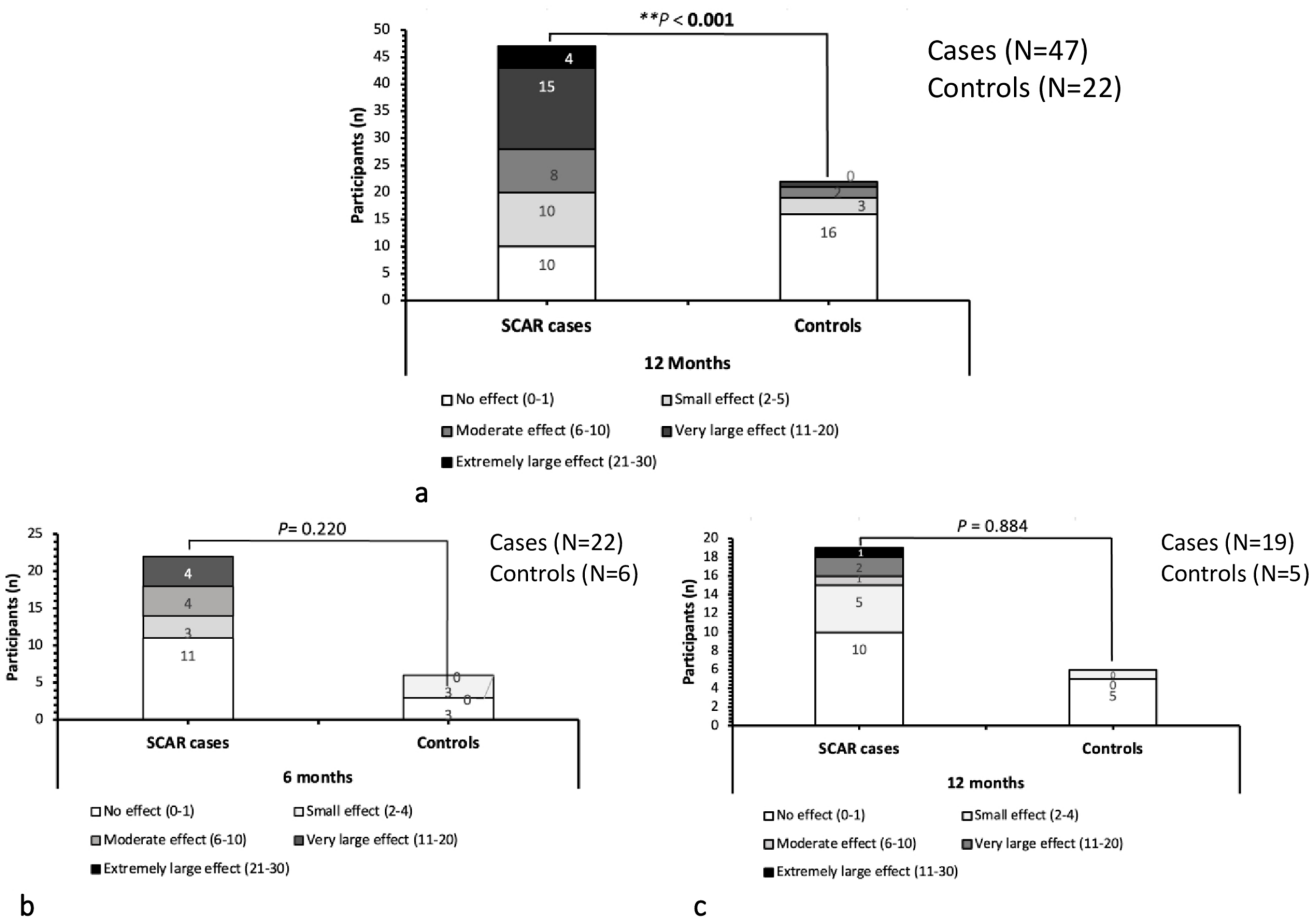
Effect of SCAR on QoL (DQLI total score observed) vs. control at baseline **(a)**; 6 months **(b)** and 12 months follow-up **(c)**; * *p*-value <0.05 = statistically significant difference between cases vs. controls outcomes at baseline, 6 months, and 12 months follow-up.

At baseline. DLQI was impaired in 27/47 (58%) of the cases and 3/22 (14%). The overall impairment in QoL (DLQI 11–20) was “very large effect” with a total median (IQR) DLQI score of 11 (5–15) among the 69 participants, 57% of cases fell into the moderate-to-severe category compared with 14% of controls. The median (IQR) DLQI score was significantly higher in cases than controls [9.3 (4–15) vs. 1.5 (0–3), *p* < 0.001] (see Figure 4a).

At 6-month follow-up, the overall impairment in QoL (DLQI 2–5) was “small effect” with a total median DLQI score of 5 (4–6). Cases demonstrated higher levels of very large’ impairment in QoL (DLQI score ≥6) compared with controls (36% vs. 0%), though the difference was not statistically significant (*p* = 0.220, Figure 4b). From baseline, 3/27 (11%) of cases had persistently and clinically abnormal DLQI scores (≥6) compared with 1/3 (33%) of the controls. Worsening was observed in 3/20 (15%) cases who did not have clinically abnormal DLQI scores (<6) verses none in the controls.

At the 12-month follow-up, four cases had abnormal DLQI scores (<6). Three of these were persistent cases from 6 months earlier and one had not been seen at 6 months and only seen again at 12 months. The overall impairment in quality of life (DLQI 2–5) was “small effect” with a total median DLQI score of 2.5 (1–10), cases versus 0 controls, although the difference was not statistically significant (*p* = 0.220, see Figure 4c).

## Discussion

This case-control study aimed to prospectively examine the longitudinal trajectories of psychological and body image sequelae in patients who develop SCARs and compare them to a control group comprising of individuals with less severe skin drug eruptions, those with dermatosis other than drug eruptions, and drug-tolerant TB and HIV patients. The study population predominantly comprised individuals with higher Fitzpatrick skin phototypes, a group that remains underrepresented in SCAR and the dermatology literature. Antibiotics and anti-tuberculosis drugs were the most commonly implicated agents in SCARs. Bactrim exposure was significantly higher among cases, whereas antiretroviral exposure was more frequent in controls, suggesting differential drug exposure and tolerance. These findings are consistent with reports from HIV- and TB-endemic settings, where sulphonamides and anti-tuberculosis drugs are major causes of SCARs, with variations across populations likely reflecting differences in prescribing patterns and genetic susceptibility (7–9, 11). These findings may reflect underlying pharmacogenetic susceptibility, as *HLA* alleles such as HLA-B*44:03, HLA-B*38:01, and HLA-C*04:01, are independently associated with co-trimoxazole-induced SJS/TEN in the United States. HLA-B*44:03 was significantly associated with co-trimoxazole-induced DRESS in South Africa (47). Particularly, HLA-B*13:01 allele has emerged as a robust pharmacogenomic marker for dapsone-induced SCARs in Thai and other Asian populations, demonstrating strong allele-drug specificity (48). In addition, HLA-B*15:02 allele was strongly associated with carbamazepine-induced SJS/TEN in Han Chinese, Thais, Vietnamese, Malaysian, and Indian populations (49). Nevertheless, HLA-A*31:01 associated with carbamazepine-induced SJS/TEN, DRESS, and maculopapular exanthema (MPE) in European, Japanese, Taiwan, Han Chinese, and Korean population (49). Moreover, previous studies showed that allopurinol-induced SJS, TEN, DRESS, and MPE were associated with HLA-B*58:01 in all populations (50). Although not assessed in this study, the predominance of antibiotics and anti-tuberculosis drugs as causative agents in our cohort highlights the need for future studies to explore genetic susceptibility in HIV- and TB-endemic settings.

The major findings of this study were as follows: (i) Nearly half of the acute SCAR cases were clinically depressed with half of them having suicidal ideation. In contrast only 1 in 10 controls were depressed, with a quarter having suicidal ideation; (ii) Depression persisted in one-fifth of SCAR patients with ongoing suicidal risk in half of them; (iii) SCARs profoundly impacted body image, with four-fifths of cases being moderate/severe on the CBI. However, these figures dropped to half and a quarter at 6 and 12 months, respectively; and (iv) SCARs acutely and persistently impaired QoL compared to controls. These findings highlight the immense psychological burden associated with SCARs, likely driven by physical sequelae such as scarring and severe pain, traumatic experiences, and the psychosocial impact of visible disfigurement such as post-inflammatory hyperpigmentation (PIPH). This further supports the growing body of evidence showing a strong link between SCARs and negative psychological outcomes (25, 32, 51, 52).

We found that cases had significantly higher odds of depression at baseline compared to controls, with the latter having adjusted odds ratio of depressive symptoms (aOR = 0.14; 95% CI, 0.03–0.55; *p* = 0.005), which aligns with previous evidence. Chiang et al. similarly demonstrated patients with SJS and TEN were at a greater risk of developing psychiatric disorders compared to matched controls. Their adjusted hazard ratios of psychiatric disorders were 1.290 (95% CI, 1.105–1.506; *p* < 0.001) for SJS and 1.855 (95% CI, 1.587–2.167; *p* < 0.001) for TEN, which represents the more severe end of the disease (51). Although the studies differ in their approach to analysis (cross-sectional evidence vs. hazard ratios), populations (HIV/TB-affected vs. general population); follow-up period, ethnicity (Africans vs. Chinese), and study design (prospective vs. retrospective), their findings correlate. Both studies indicate that SCARs are associated with elevated vulnerability to depression. This convergence strengthens the evidence base and emphasizes the importance of routine psychological screening and support in SCAR care. Length of admission was not meaningfully associated with the risk of developing depression. Although each additional day of hospitalization was linked to a 1% reduction in the odds of depression (aOR = 0.99; 95% CI, 0.98–1.01; *p* = 0.53), this association was small in magnitude and not statistically significant.

This study found that SCAR patients had significantly lower CBI scores (<6) compared to controls at baseline (80% vs. 56%, *p* = 0.023), confirming a more negative perception of their bodies. CBI scores remained lower among cases than controls at 6 months (*p* = 0.376) and 12 months (*p >* 0.9), demonstrating a sustained difference across all time points. This highlights the enduring negative impact of SCARs on body image perception, even months after the acute event. Additionally, 9% of cases worsened, transitioning from moderate to severe. One case improved, while one case and one control relapsed at 12 months. In contrast, these patterns were not observed among controls. These findings highlights the lasting esthetic impact of SCARs, which extends beyond physical recovery. The low CBI scores among the cases may reflect the visible sequelae of SCARs, including scarring, changes in pigmentation, nail changes, and ocular/mucosal damage, which are known to negatively influence on both self-perception and social functioning (53–55). To our knowledge, this is the first study to assess CBI in SCARs and demonstrate negative body image perception. The significant difference in CBI scores between cases and controls emphasize the importance of routine assessment of body image disturbance in SCAR survivors. Identifying patients at risk of poor self-perception could guide referral for psychological support and counseling. This further emphasizes that SCARs should be regarded not only as a dermatological emergency but also as a condition with significant long-term psychosocial consequences that require integrated, multidisciplinary approach and follow-up. Dedicated drug allergy clinics, bringing together dermatologists, pharmacologists, psychologists, psychiatrists, and immunologists to provide comprehensive care for SCAR survivors would go a long way to improve outcomes. Non-genetic factors that may increase susceptibility to SCARs include underlying comorbidities (such as HIV, tuberculosis, renal impairment, and autoimmune diseases), female sex, older age, and high initial doses of high-risk drugs such as anticonvulsants and allopurinol (30, 56). Infectious triggers also contribute, including reactivation of herpesviruses and Mycoplasma pneumoniae, the latter being a recognized non-drug cause of SJS/TEN, particularly in children (57–59). However, these findings indicated no association between HIV/TB and the outcome of depression in this cohort. More studies with a large sample size are needed to validate the findings.

We found that patients with SCARs reported lower quality of life compared to controls across multiple time points. The majority of existing studies have been cross-sectional or retrospective, documenting persistent physical, psychological, and social sequelae; however, they have not prospectively followed patients alongside matched controls. For example, Hoffman et al. (32) and Yang et al. (35) described substantial long-term impairments in QoL, including anxiety, depression, and physical limitations, yet their designs did not include control groups or repeated measures over time. Similarly, qualitative work from the earlier studies, highlighted profound impacts on QoL but again lacked longitudinal quantitative comparisons controls (25). Our findings align with those of Hoffman et al., who described long-lasting functional and psychosocial limitations on SCAR survivors, while Yang et al. reported persistent impairment in multiple QoL domains even years after the acute episode (32, 35). Similarly, a qualitative study by O’Reilly et al. highlighted ongoing distress and negative self-perceptions well beyond hospital discharge (34).

These data support a significant long-term psychological burden of SCARs, extending beyond just the acute stages and demonstrating a more pronounced worsening trajectory. This may reflect differences in patient demographics, severity of sequelae, or the absence of structured long-term psychosocial support. There is a need for multicenter prospective cohorts to validate our findings, to determine whether trajectories improve, persist, or worsen over time, and to identify modifiable factors that could guide supportive interventions. The significance of this study extends beyond academic interest. It has the potential to initiate collaborative epidemiological effort within and among low-to-middle income countries to better understand mental health disorders in IM-ADRs. This research could influence public health policies and healthcare practices, ultimately improving the lives of HIV/TB patients and others affected by SCAR-associated psychological sequelae.

Difference in attrition rates between cases and controls was observed at 6 months, introducing potential attrition bias. However, no significant differences were identified in baseline demographic or clinical characteristics between participants retained and those lost to follow-up. These findings suggest that attrition was unlikely to be strongly related to disease severity. Nevertheless, residual survivor bias cannot be excluded and should be considered when interpreting longitudinal comparisons.

In addition, the substantial loss to follow-up coincided with periods of COVID-19-related healthcare disruption, which may have contributed to non-differential attrition. Reduced clinic accessibility and patient reluctance to attend in-person visits likely contributed to attrition. Although baseline demographic characteristics did not differ between retained and non-retained participants, residual attrition bias related to unmeasured factors (such as socioeconomic factors) cannot be excluded. We believe that these additional analyses strengthen the robustness of our findings.

### Limitations

COVID-19 lockdowns and associated restricted travel and clinic closures caused greater than expected losses to follow-up, particularly in the control group, limiting conclusions regarding longitudinal effects. However, attrition was comparable between cases and controls, with no differences in key baseline characteristics (age, gender, or disease severity) between participants who completed the study and those who were lost to follow-up. The MINI, the Kessler Psychological Distress Scale (K10), and the CBI questionnaire have not been formally validated in HIV/TB-infected African populations, and cultural, linguistic, and disease-related factors may have influenced participants’ responses. However, we have successfully used these instruments in previous studies conducted in the South African context, and the results have been consistent. This suggests that despite these cultural and linguistic challenges, the instruments perform well. Therefore, while the reduced sample size may limit the generalizability of the findings, the final cohort is considered broadly representative of the initial study population. There were no significant differences between the included and lost-to-follow-up patient characteristics.

## Conclusion

SCARs were found to be associated with a high prevalence of depression and suicide risk irrespective of HIV or TB status, and were significantly associated with both impaired QoL and severe negative self-perception compared to controls. SCAR patients demonstrated persistent or worsening impairments at 6 months and relapses at 12 months. These findings highlight the ongoing psychosocial burden of SCARs and the need for integrated mental health support.

## Data Availability

The original contributions presented in the study are included in the article/Supplementary material, further inquiries can be directed to the corresponding author.
