# Supplementary material for: Prospective longitudinal study of psychological sequelae, self-perception of body image, and quality of life in severe cutaneous adverse drug reactions: a case-control study
Source: Front Med (Lausanne). 2026 May 29;13:1774494. doi: 10.3389/fmed.2026.1774494 (PMC13259666; doi:10.3389/fmed.2026.1774494)
Supplement: Supplementary file 1 [file Table_1.DOCX]

**Supplementary table 1 (S1).** Summary of participant recruitment and retention by study group (cases vs. controls)

| **Participant Category** | **Cases (n)** | **Controls (n)** | **Total (n)** |
| --- | --- | --- | --- |
| **Initially Enrolled** | 124 | 89 | **213** |
| **Excluded due to eligibility criteria** | 47 | 35 | **82** |
| **Lost to Follow-up** | 30 | 24 | **54** |
| **Final analysed sample** | **47** | **30** | **77** |
| Retention rate | 47/124 = **37.9%** | 30/89 = **33.7%** | 77/213 = **36.1%** |
