# Supplementary material for: Prospective longitudinal study of psychological sequelae, self-perception of body image, and quality of life in severe cutaneous adverse drug reactions: a case-control study
Source: Front Med (Lausanne). 2026 May 29;13:1774494. doi: 10.3389/fmed.2026.1774494 (PMC13259666; doi:10.3389/fmed.2026.1774494)
Supplement: Supplementary file 2 [file Table_2.DOCX]

**Supplementary table 2 (S2).** Logistic regression assessing the odds to lost to follow-up (attrition) at 6 months and 12 months adjusted for age, gender, case and control, and baseline depression.

| **Follow-up status** | **Variables** | **Odds ratio** | **95% Conf. Interval** | ***P* = value** |
| --- | --- | --- | --- | --- |
| **6 months** |  |  |  |  |
|  | Age | 1.02 | 0.98-1.07 | 0.300 |
|  | Gender | 2.05 | 0.68-6.22 | 0.203 |
|  | Case_controls | 3.72 | 1.18-11.66 | **0.024*** |
|  | Baseline depression | 0.65 | 0.21-1.92 | 0.438 |
| **12 months** |  |  |  |  |
|  | Age | 1.01 | 0.97-1.05 | 0.588 |
|  | Gender | 0.94 | 0.31-2.78 | 0.914 |
|  | Case_controls | 2.66 | 0.85-8.34 | 0.093 |
|  | Baseline depression | 1.65 | 0.52-5.19 | 0.391 |
| ******P*-value < 0.05 = statistically significant between variables. | | | | |
