# Supplementary material for: Prospective longitudinal study of psychological sequelae, self-perception of body image, and quality of life in severe cutaneous adverse drug reactions: a case-control study
Source: Front Med (Lausanne). 2026 May 29;13:1774494. doi: 10.3389/fmed.2026.1774494 (PMC13259666; doi:10.3389/fmed.2026.1774494)
Supplement: Supplementary file 3 [file Table_3.DOCX]

**Supplementary table 3 (S3).** Sensitivity analysis assessing the association between case status and control subgroup (those with and those without skin disease).

| **3a** | | | |
| --- | --- | --- | --- |
| **Baseline Depression** | **Cases (n=47)** | **Controls (those without skin disease) (n =8)** | ***P* =value** |
| - Yes, n(%) | 21 (44.68) | 1 (12.50) | 0.086*^1^* |
| - No, n(%) | 26 (55.32) | 7 (87.50) |  |
| **3b** | | | |
| **Baseline Depression** | **Cases (n=47)** | **Controls (those with skin disease) (n =22)** | ***P* =value** |
| - Yes, n(%) | 21 (44.68) | 3 (13.64) | 0.012****^,1^*** |
| - No, n(%) | 26 (55.32) | 19 (86.36) |  |
| **n (%)** – number of participants and percentages; ***^1^***The Chi-square; ******P*-value < 0.05 = statistically significant between cases vs. controls. | | | |
