# Supplementary material for: Prospective longitudinal study of psychological sequelae, self-perception of body image, and quality of life in severe cutaneous adverse drug reactions: a case-control study
Source: Front Med (Lausanne). 2026 May 29;13:1774494. doi: 10.3389/fmed.2026.1774494 (PMC13259666; doi:10.3389/fmed.2026.1774494)
Supplement: Supplementary file 4 [file Table_4.DOCX]

**Supplementary table 4 (S4). Raw data for all participants enrolled in the study.**

| **Study #** | **Participant #** | **Case (1) /Control(2)** | **Admission day** | **Discharge day** | **Admission days** | **DOB** | **AGE** | **Phenotype** | **BSA of rash** | **Offending Drug** | **Gender** | **Ethnicity** | **HIV** | **CD4 count** | **TB** | **Type of TB** |
| --- | --- | --- | --- | --- | --- | --- | --- | --- | --- | --- | --- | --- | --- | --- | --- | --- |
| 139 | 1 | 1 | 27/01/21 | 05/02/21 | 9 | 26/12/1967 | 53 | DRESS | 60% | Bactrim | Female | Black | Positive | 86 | Yes | Pulmonary |
| 144 | 2 | 1 | 12/2/2021 | 10/3/2021 | 29 | 17/7/1980 | 31 | DRESS | 95% | RHZE as Rifafour | Female | Black | Negative | N/A | Yes | Pulmonary |
| 162 | 3 | 1 | 21/04/21 | 07/05/21 | 16 | 22/8/1084 | 37 | DRESS | 60% | Rifabutin, Ethambutol | Female | Black | Positive | 36 | yes | Dis-TB |
| 164 | 4 | 1 | 12/05/11 | 21/05/21 | 9 | 10/02/1988 | 33 | DRESS | 10% | Bactrim | Male | Colored | Positive | 167 | Yes | Dis-TB |
| 165 | 5 | 1 | 2021/05/18 | 2021/07/23 | 66 | 1968-05-04 | 33 | DRESS | 60% | Bactrim | Female | Colored | Positive | 82 | Yes | Dis-TB |
| 188 | 6 | 1 | 27/09/21 | 25/10/21 | 28 | 02/08/1987 | 34 | DRESS | 10% | Rifampicin | Female | Black | Negative |  | Yes | Dis-TB |
| 192 | 7 | 1 | 6/10/2021 | 18/10/2021 | 12 | 1970-11-04 | 51 | DRESS | 55% | Carbamazepine | Female | Colored | Negative | N/A | No | N/A |
| 197 | 8 | 1 | 2021/10/18 | 08/11/2021 | 21 | 1971-01-02 | 50 | DRESS | 40% | Bactrim | Female | Colored | Positive | 111 | Yes | Pulmonary |
| 198 | 10 | 1 | 2021/10/20 | 03/01/2022 | 75 | 1978-06-15 | 43 | DRESS | 95% | RHZE as Rifafour | Male | Black | Positive | 90 | Yes | Dis-TB |
| 216 | 11 | 1 | 2021/12/13 | 16/01/2022 | 34 | 1977-10-15 | 44 | DRESS | 60% | RHZE as Rifafour | Female | Black | Positive | 115 | Yes | Pulmonary |
| 219 | 12 | 1 | 2022/01/03 | 22/02/2022 | 50 | 1967-01-07 | 55 | DRESS | 80% | RHZE as Rifafour | Male | Colored | Positive | 197 | Yes | Dis-TB |
| 238 | 13 | 1 | 08/04/22 | 26/04/22 | 18 | 09/12/1964 | 57 | DRESS | 90% | Bactrim, PZA, EMB | Male | Black | Positive | 119 | Yes | Pulmonary |
| 243 | 14 | 1 | 09/05/22 | 25/05/22 | 16 | 15/09/1979 | 42 | DRESS | 80% | Bactrim, PZA, EMB | Male | Black | Positive | 111 | Yes | Dis-TB |
| 245 | 15 | 1 | 12-05-2022 | 20-05-2022 | 8 | 09/03/1982 | 40 | DRESS | 50% | Bactrim | Female | Colored | Positive | 138 | Yes | Pulmonary |
| 263 | 16 | 1 | 27/07/22 | 28/09/22 | 63 | 09/03/1992 | 30 | DRESS | 85% | Levofloxacin, , | Female | Black | Positive | 103 | Yes | Dis-TB |
| 266 | 17 | 1 | 06/08/22 | 15/08/22 | 9 | 04/06/1963 | 59 | DRESS | 90% | Bactrim | Male | Black | Positive | 93 | Yes | Pulmonary |
| 267 | 18 | 1 | 08/08/22 | 19/10/22 | 72 | 13/05/1994 | 28 | DRESS | 80% | PZA (by exclusion) | Male | Colored | Positive | 238 | Yes | Pulmonary |
| 279 | 19 | 1 | 31/08/22 | 09/09/22 | 9 | 27/04/1986 | 36 | DRESS | 85% | RHZE | Female | Black | Positive | 227 | Yes | Dis-TB |
| 280 | 20 | 1 | 07/09/2022 | 07/10/022 | 30 | 1983-12-25 | 39 | DRESS | 85% | Sulfasalazine | Female | Black | Negative | N/A | No | N/A |
| 281 | 21 | 1 | 2022/08/19 | 26/09/2022 | 38 | 1987-09-07 | 35 | DRESS | 95% | Bactrim, PZA, EMB | Female | Black | Positive | 331 | Yes | Pulmonary |
| 318 | 22 | 1 | 2022/11/22 | 24/09/2022 | 59 | 1976-01-31 | 46 | DRESS | 75% | Bactrim, Dapsone | Female | Black | Positive | 64 | No | N/A |
| 322 | 23 | 1 | 01/12/22 | 23/12/22 | 22 | 01/10/1992 | 30 | DRESS | 10% | Rifampicin | Female | Black | Positive | 142 | Yes | Pulmonary |
| 323 | 24 | 1 | 05/12/22 | 23/12/22 | 18 | 26/06/1985 | 36 | DRESS | 90% | Bactrim | Female | Black | Positive | 137 | Yes | TBM |
| 333 | 25 | 1 | 11/01/23 | 20/02/23 | 40 | 28/09/1995 | 27 | DRESS | 60% | RHZE as RIFAFOUR | Female | Black | Positive | 21 | Yes | Pulmonary |
| 347 | 26 | 1 | 20/02/23 | 27/02/23 | 7 | 29/11/1984 | 38 | DRESS | 95% | Rifampicin, PZA | Female | Black | Positive | 394 | Yes | Pulmonary |
| 360 | 27 | 1 | 26/04/2023 | 24/05/2023 | 28 | 2002-09-19 | 21 | DRESS | 90% | RHZE or Bactrim | Female | Black | Positive | 211 | Yes | Pulmonary |
| 422 | 28 | 1 | 2023/10/10 | 27/10/2023 | 17 | 1960-04-04 | 63 | DRESS | 20% | Terbinafine | Male | White | Negative | N/A | No | N/A |
| 426 | 29 | 1 | 2023/10/18 | 27/10/2023 | 21 | 1948-10-01 | 75 | DRESS | 80% | Allopurinol | Male | White | Negative | N/A | No | N/A |
| 430 | 30 | 1 | 2023/10/23 | 10/11/2023 | 18 | 1989-02-11 | 34 | DRESS | 80% | Lamotrigine | Female | Black | Negative | N/A | No | N/A |
| 438 | 31 | 1 | 2023/11/06 | 30/11/2023 | 24 | 1960-06-11 | 63 | DRESS | 60% | Carbamazepine, | Female | Indian | Negative | N/A | No | N/A |
| 445 | 32 | 1 | 2023/11/27 | 04/12/2023 | 7 | 1989-07-05 | 34 | DRESS | 70% | CT contrast | Female | Colored | Negative | N/A | No | N/A |
| 453 | 33 | 1 | 2024/01/02 | 31/01/2024 | 29 | 1984-07-21 | 40 | DRESS | 40% | Bactrim, RHZE | Male | Black | Positive | 155 | Yes | Dis-TB |
| 458 | 34 | 1 | 2024/01/22 | 2024/01/13 | 9 | 1964-06-29 | 60 | DRESS | 75% | Bactrim | Male | White | Negative | N/A | No | N/A |
| 679-90 | 35 | 1 | 2023/12/12 | 12-Dec-23 | 0 | 1965-01-01 | 58 | DRESS | 36% | Ertapenem | Male | Black | Negative | N/A | No | N/A |
| 679-94 | 36 | 1 | 2024/03/08 | 18/03/2024 | 10 | 1980-05-07 | 44 | DRESS | 80% | NSAIDS | Female | Black | Negative | N/A | No | N/A |
| 191 | 37 | 1 | 16/09/2021 | 17/11/2021 | 62 | 1970-01-01 | 51 | SJS/TEN | 40% | Rifampicin | Male | Black | Positive | 23 | Yes | Dis-TB |
| 220 | 38 | 1 | 17/01/2022 | 09/02/2022 | 23 | 10/12/1976 | 46 | SJS/TEN | 85% | Bactrim | Male | Black | Positive | 9 | Yes | Dis-TB |
| 300 | 39 | 1 | 22/10/2022 | 13/12/2022 | 82 | 07/09/1972 | 50 | SJS/TEN | 95% | Rifampicin | Male | Black | Positive | 33 | Yes | Dis-TB |
| 343 | 40 | 1 | 11/01/2023 | 20/02/2023 | 40 | 02/09/1994 | 29 | SJS/TEN | 60% | RHZE as Rifafour | Female | Black | Positive | 21 | Yes | Pulmonary |
| 383 | 41 | 1 | 07/07/2023 | 21/07/2023 | 14 | 22/08/1992 | 31 | SJS/TEN | 70% | Bactrim | Female | Black | Positive | 145 | Yes | Pulmonary |
| 425 | 42 | 1 | 17/10/2023 | 06/11.2023 | 50 | 20/11/1995 | 28 | SJS/TEN | 40% | Bactrim | Male | Black | Positive | 5 | Yes | Pulmonary |
| 679-91 | 43 | 1 | 13/12/2023 | 18/12/2023 | 5 | 03/04/1959 | 64 | SJS/TEN | 30% | Bactrim | Male | Black | Positive | 115 | Yes | Pulmonary |
| 679-92 | 44 | 1 | 05/02/2024 | 15/03/2024 | 39 | 10/08/1980 | 44 | SJS/TEN | 80% | Bactrim | Male | Black | Positive | 92 | Yes | Pulmonary |
| 207 | 45 | 1 | 10/11/2021 | 19/11/2021 | 9 | 20/06/1972 | 49 | GBFDE | 10% | Bactrim | Female | Black | Positive | 588 | No | N/A |
| 288 | 46 | 1 | 30/09/2022 | 10/10/2022 | 10 | 08/12/1985 | 37 | GBFDE | 15% | Bactrim | Female | Colored | Positive | 66 | No | N/A |
| 346 | 47 | 1 | 18/01/2023 | 13/03/2023 | 54 | 09/05/1980 | 43 | GBFDE | 35% | PZA | Male | Black | Positive | 181 | Yes | Pulmonary |
| 451 | 48 | 1 | 04/01/2024 | 31/01/2024 | 27 | 20/06/1988 | 36 | GBFDE | 2% | RHZE | Female | Black | Positive | 538 | Yes | Dis-TB |
| 13 | 49 | 2 | 18/01/2019 | 27/03/2019 | 69 | 25-Jan-95 | 24 | Control | 18% | Efavirenz | Female | Black | Positive | 778 | No | N/A |
| 123 | 50 | 2 | 03/07/2020 | 03/07/2020 | 0 | 13-Dec-66 | 54 | Control | 60% | Bactrim | Female | Black | Negative | N/A | Yes | Pulmonary |
| 170 | 51 | 2 | 24/05/2021 | 02/06/2021 | 9 | 13-Nov-97 | 24 | Control | 15% | Bactrim | Female | Black | Negative | N/A | No | N/A |
| 171 | 52 | 2 | 25/05/2021 | 25/05/2021 | 0 | 20-Dec-97 | 24 | Control | 60% | Bactrim | Female | Black | Positive | 43 | No | N/A |
| 177 | 53 | 2 | 14/07/2021 | 04/07/2021 | 21 | 21-Apr-87 | 34 | Control | 55% | Bactrim | Female | Black | Positive | 22 | Yes | Pulmonary |
| 206 | 54 | 2 | 08/11/2021 | 16/11/2021 | 8 | 14-Mar-77 | 44 | Control | 25% | Rifampicin | Male | Colored | Negative | N/A | Yes | Dis-TB |
| 217 | 55 | 2 | 26/12/2021 | 14/01/2022 | 19 | 09-Jul-62 | 59 | Control | 80% | Ciprobay | Male | Colored | Positive | 58 | No | N/A |
| 229 | 56 | 2 | 24/02/2022 | 02/03/2022 | 6 | 05-Feb-46 | 76 | Control | 2% | RHZE as Rifafour | Female | Black | Negative | N/A | No | N/A |
| 277 | 57 | 2 | 04/08/2022 | 14/09/2022 | 41 | 27-Apr-92 | 30 | Control | 40% | RHZE as Rifafour | Female | Black | Negative | N/A | Yes | Pulmonary |
| 278 | 58 | 2 | 05/09/2022 | 21/09/2022 | 16 | 23-Jan-96 | 26 | Control | 85% | RHZE as Rifafour | Female | Black | Positive | 26 | Yes | Pulmonary |
| 286 | 59 | 2 | 01/10/2021 | 10/10/2022 | 374 | 22-Dec-01 | 20 | Control | 80% | RHZE as Rifafour | Male | Black | Positive | 82 | No | N/A |
| 324 | 60 | 2 | 16/11/2022 | 16/11/2022 | 0 | 29-Jan-56 | 66 | Control | 30% | RHZE as Rifafour | Male | White | Negative | N/A | Yes | TBM |
| 327 | 61 | 2 | 15/11/2022 | 28/02/2023 | 105 | 17-Dec-89 | 33 | Control | 90% | RHZE as Rifafour | Female | Colored | Positive | 50 | Yes | Dis-TB |
| 334 | 62 | 2 | 01/03/2022 | 01/03/2022 | 0 | 15-Feb-95 | 27 | Control | 90% | ARV's | Female | Black | Positive | 771 | No | N/A |
| 335 | 63 | 2 | 23/01/2023 | 31/01/2023 | 8 | 27-Jan-78 | 45 | Control | 90% | RHZE as Rifafour | Female | Black | Positive | 228 | Yes | Dis-TB |
| 361 | 64 | 2 | 04/04/2023 | 04/04/2023 | 0 | 14-Aug-60 | 63 | Control | 0% | RHZE as Rifafour | Male | Black | Positive | 19 | Yes | Pulmonary |
| 382 | 65 | 2 | 14/06/2023 | 14/06/2023 | 0 | 05-Apr-88 | 35 | Control | 20% | Bactrim | Female | Black | Positive | 136 | Yes | Pulmonary |
| 419 | 66 | 2 | 09/10/2023 | 16/10/2023 | 7 | 18-Oct-99 | 24 | Control | 15% | Bactrim | Female | Colored | Positive | 377 | Yes | Pulmonary |
| 428 | 67 | 2 | 21/10/2023 | 23/10/2023 | 2 | 02-Oct-69 | 54 | Control | 10% | N/A | Female | Colored | Negative | N/A | No | N/A |
| 442 | 68 | 2 | 21/11/2023 | 27/11/2023 | 6 | 01-Aug-53 | 70 | Control | 70% | N/A | Male | Colored | Negative | N/A | No | N/A |
| 450 | 69 | 2 | 14/12/2023 | 23/12/2023 | 9 | 26-Jul-74 | 53 | Control | 70% | RHZE as Rifafour | Female | Black | Positive | 233 | Yes | Dis-TB |
| 459 | 70 | 2 | 19/01/2024 | 19/01/2024 | 0 | 08-Feb-77 | 47 | Control | 30% | N/A | Female | Black | Positive | 315 | Yes | Pulmonary |
| 679-1 | 71 | 2 | 03/03/2021 | 26/03/2021 | 23 | 12-Oct-75 | 46 | Control | 5% | N/A | Female | Black | Positive | 14 | No | N/A |
| 70 | 72 | 2 | 27/08/2019 | 28/08/2019 | 0 | 05-Dec-88 | 31 | Control | 0% | RHZE as Rifafour | Male | Black | Positive | 158 | Yes | Pulmonary |
| 156 | 73 | 2 | 20/04/2021 | 20/04/2021 | 0 | 21-Jan-85 | 36 | Control | 0% | ARV's | Male | Black | Positive | 300 | No | N/A |
| 157 | 74 | 2 | 20/04/2021 | 21/04/2021 | 0 | 06-Nov-85 | 36 | Control | 0% | ARV's | Male | Black | Positive | 45 | No | N/A |
| 158 | 75 | 2 | 20/04/2021 | 21/04/2021 | 0 | 05-Apr-70 | 51 | Control | 0% | ARV's | Female | Black | Positive |  | No | N/A |
| 159 | 76 | 2 | 20/04/2021 | 21/04/2021 | 0 | 25-Oct-68 | 53 | Control | 0% | ARV's | Female | Colored | Positive | 601 | No | N/A |
| 160 | 78 | 2 | 20/04/2021 | 21/04/2021 | 0 | 23-Aug-87 | 34 | Control | 0% | ARV's | Male | Black | Positive | 242 | No | N/A |
| 161 | 79 | 2 | 20/04/2021 | 21/04/2021 | 0 | 07-Jun-87 | 34 | Control | 0% | ARV's | Male | Colored | Positive | 1497 | No | N/A |
| **#** = number**, DOB** = Date of birth, **BSA** = Body surface area, **HIV** = Human Immune virus, **TB** = Tuberculosis, **DRESS** = Drug rash eosinophilia and systemic symptoms syndrome , **SJS/TEN** = epidermal necrolysis, **GBFDE** = Generalized bullous fixed drug eruption , **RHZE** = Rifafour TB medication (rifampicin, Isoniazid, Pyrazinamide and Ethambutol), **Dis-TB** = Disseminated -Tb, **TBM** = Tb -Meningitis, **ARV's** = Antiretrovirals treatment, **N/A** = not captured. | | | | | | | | | | | | | | | | |
